# Busy day effect on intrapartum adverse maternal outcomes – a population‐based study of 601 247 singleton deliveries

**DOI:** 10.1186/s12884-021-03552-8

**Published:** 2021-01-19

**Authors:** Riitta Vilkko, Sari Räisänen, Mika Gissler, Vedran Stefanovic, Seppo Heinonen

**Affiliations:** 1 Doctoral Programme in Clinical Research, Faculty of Medicine, University of Helsinki, Haartmanninkatu 8, 00290 Helsinki, Finland; 2 School of Health, Tampere University of Applied Sciences, Kuntokatu 3, 33520 Tampere, Finland; 3 Information Services Department, THL Finnish Institute for Health and Welfare, Mannerheimintie 166, 00270 Helsinki, Finland; 4 Department of Obstetrics and Gynecology, Fetomaternal Medical Center, Helsinki University Hospital and University of Helsinki, Haartmaninkatu 2, 00290 Helsinki, Finland; 5 Department of Obstetrics and Gynecology, Helsinki University Hospital and University of Helsinki, Haartmaninkatu 2, 00290 Helsinki, Finland

**Keywords:** Adverse maternal outcome, Blood transfusion, Busy day effect, Daily delivery volume, Hospital size, Medical birth register

## Abstract

**Background:**

This was a retrospective population-based study, utilizing the data of 601 247 singleton hospital deliveries collected from the Finnish Medical Birth Register (MBR) in 2006–2016. The aim of this study was to analyse the busy day effect on intrapartum adverse maternal outcomes.

**Methods:**

To implement the study design, daily delivery frequencies and ranges (min-max) for each delivery unit (*n* = 26) were stratified to the daily delivery volume distributions by the delivery unit’s annual delivery volume and profile: Category (C)1 < 1000, C2 1000–1999, C3 2000–2999, C4 ≥ 3000 and C5 the profile of university hospitals. To study the busy day effect, the quiet, optimal and busy days were defined by calculating the number of days (%) with the lowest and highest daily delivery frequencies and summed to the nearest 10 % in each hospital category. Optimal days were determined by calculating approximately 80 % of deliveries occurring between the lowest 10 %, and highest 10 % in each hospital category. Crude and adjusted odd ratios (ORs) with 99 % confidence intervals (CIs) were used to analyze the busy day effect on adverse maternal outcomes, blood transfusions, manual removal of the placenta and obstetric anal sphincter injuries, separately in each hospital category.

**Results:**

The busy day effect was associated with the 28 % (99 % CI 8–52 %) and 25 % (99 % CI 11–40 %) increased need for blood transfusions in C2 and university hospitals (C5), respectively, whereas 22 % (99 % CI 10–31 %) less blood transfusions were needed at university hospitals during quiet days. In C3 hospitals, 83 % (99 % CI 65–92 %) less blood transfusions were needed during busy days. Obstetric and anal sphincter injury rates declined during quiet days by 22 % (99 % CI 3–38 %) only in university hospitals.

**Conclusions:**

The findings of this study identify no specific pattern to the busy day effect for adverse maternal outcomes defined as manual removal of the placenta or obstetric and anal sphincter injuries. However, both quiet and busy days seem to be associated with increased or decreased need for blood transfusions in different sized delivery units. Findings also suggest that quiet days are associated with a decreased number of obstetric and anal sphincter injuries.

## Background

Giving birth is an individual and often unpredictable process. Elective Caesarean is the only delivery mode which can be scheduled beforehand. The nature of the delivery process causes challenges to delivery unit organisations. Daily inconsistent occurrences and varying patient flow in delivery units can cause a range from quiet to busy time periods compared to unit´s optimal period in terms of capacity and staffing. Delivery unit organisations have a responsibility to ensure patient safety and keep the balance in quality of care despite of these daily changes in patient flow [1]. We have shown in the earlier studies the occurrence of daily delivery volume changes in particular within small delivery units (< 1000 annual deliveries), where the variation of daily patient flow is more prominent compared to the larger ones (Vilkko R, Räisänen S, Stefanovic V, Gissler M, Heinonen S: Patient flow unevenness in different sized delivery hospitals – an 11-year register study of 610 227 deliveries, unpublished). This implies that hospital capacity may become overloaded and have an effect on the delivery unit practises. As a consequence, earlier results also indicate that busy days tend to produce excessive interventions during labour (Vilkko R, Räisänen S, Gissler M, Stefanovic V, Heinonen S: Busy day effect on the use of interventions during labour – A population-based register study of 601 247 singleton deliveries, unpublished). For these reasons, busy days in delivery units can be seen as a risk factor for adverse maternal outcomes. However, the other existing evidence on this is limited since quality indicators [2] are more often assessed and based on annual or monthly statistics [3–8] or hospital level acuteness [9] or even time of the day [10, 11]. The busy day effect on maternal outcomes is a less studied topic. To understand the busy day effect, it is possible to collect hospital level quality markers on a daily basis for outcome measures which are easy to identify, occur frequently enough and vary sufficiently among hospitals [12]. In this study setting, varying patient flow days have been defined as quiet, optimal and busy and have set these definitions as exposures to select adverse maternal outcome measures as blood transfusions, manual removal of the placenta (MRP) and obstetric anal sphincter injuries (OASIS). Daily delivery frequencies and ranges (min-max) were calculated for each delivery unit and then stratified by the delivery unit´s annual delivery volume and profile to make a hospital level comparisons possible. The aim of this study is to analyse the busy day effect on selected intrapartum maternal outcomes in five categories of delivery units with different annual delivery volumes or profiles.

## Methods

This study was conducted in Finland, a Nordic country with 5.5 million inhabitants and publicly funded free of charge maternity care services used by 99 % of pregnant women. Private delivery services are not available. The data for this retrospective population-based study included 601 247 singleton deliveries from 26 delivery hospitals in 2006─2016. Multiple pregnancies (*n* = 9149) and deliveries (*n* = 24 414) occurring in delivery units (*n* = 8), which were closed because of the low annual delivery volume during the study period, were excluded. Data were gathered from the Finnish Medical Birth Register (MBR). The MBR data include all diagnoses (defined by International Statistical Classification of Diseases and Related Health Problems, ICD-10) [13] and outcomes concerning live births and stillbirths with birth weight of ≥ 500 g or gestational age ≥ 22 weeks from the beginning of pregnancy until the first seven days after birth. The Finnish national data protection legislation guided study process and authorization to use the data was given by the MBR keeper, the Finnish Institute of Health and Welfare (references: THL/1749/5.05.00/2011, THL/998/5.05.00/2013 and THL/876/5.05.00/2017).

In this study setting, to analyse the busy day effect, daily delivery frequencies and ranges (min-max) were first calculated for each of the 26 delivery units and then the daily delivery volume distribution was stratified by hospitals’ annual delivery volume or profile into five categories (C1-C5). The category C1 included the smallest local and central level delivery units (*n* = 7) across the country with annual delivery volumes of less than 1000 deliveries. The low daily and annual delivery volumes in these small delivery units resulted in a small sample size of deliveries in C1. The limit of < 1000 annual deliveries was chosen because it is aligned with Finnish legislation. In Finland, a delivery unit should have at least 1000 annual deliveries to maintain and ensure patient safety. Hospital categories C2 (*n* = 10) and C3 (*n* = 2) included local and central level delivery units with annual delivery volumes from 1000 to 1999 and from 2000 to 2999, respectively. The hospital category C4 included two large sized central level delivery units, placed near by the capital area with ≥ 3000 annual deliveries (Table 1). University hospitals in hospital category C5 were categorised as its own category, because of the profile of tertiary level care, a country wide referral system and a different patient mix compared to large central hospitals. University hospitals are focusing on demanding cases and specialist level care in the field of obstetrics. The largest university hospital is located in the capital area and it is validated to treat the most high-risk pregnancies and deliveries and take care of the most extreme preterm deliveries across country. Some delivery units may have changed from the original hospital category during the study period, however, such changes also affect the variation and keeps the analysis consistent. The type of hospital categorisation in this study setting is in line with the Finnish organisation of delivery units and allocates an adequate number of hospitals in each hospital category to provide statistical power to the analyses.

**Table 1:** Hospital categorisation, number of deliveries (n, %, mean), number and range of deliveries during defined quiet, optimal and busy days in different sized hospital categories (C1-C5)

Total number of deliveries				Quiet day		Optimal day		Busy day	
Hospital category	Hospitals/ category (n)	Deliveries (n, %)	Deliveries/day (mean)	Number (n, %) and range of daily deliveries (n)		Number (n, %) and range of daily deliveries (n)		Number (n, %) and range of daily deliveries (n)	
C1	7	55 448 (9.2)	2.0	7 212 (13.0)	1	44 056 (79.5)	2–5	4 180 (7.5)	6–10
C2	10	165 573 (27.5)	4.6	14 200 (8.6)	1–2	136 711 (82.6)	3–8	14 662 (8.9)	9–16
C3	2	54 574 (9.1)	4.5	5 303 (9.7)	1–4	42 698 (78.2)	5–11	6 573 (12.0)	12–24
C4	2	108 254 (18.0)	13.5	9 731 (9.0)	1–8	88 156 (81.4)	9–23	10 367 (9.6)	24–34
C5	5	217 398 (36.2)	10.8	24 613 (11.3)	1–7	171 148 (78.7)	8–18	21 637 (10.0)	19–30
Total	26	601 247 (100.0)	7.1	61 059 (10.2)	1–8	482 769 (80.3)	2–23	57 419 (9.5)	6–34

C1 = < 1000 deliveries annually, C2 = 1000–1999 deliveries annually, C3 = 2000–2999 deliveries annually, C4 = ≥ 3000 deliveries annually, C5 = University hospitals

The varying daily delivery volume periods were defined based on annual delivery volume as quiet, optimal and busy days by counting the daily delivery frequency and range (min-max) for each hospital category. Mean and range (min-max) of daily delivery volume varied between hospital categories, and each hospital category had its own definition for daily delivery volume variation. To define busy days, the number of days (%) with most high daily delivery volumes were summed to represent the closest 10 % on each hospital category. Quiet days were also defined by summing the number of days (%) with the most low delivery volume frequency days to the nearest 10 % in each the hospital category (C1─C5). Optimal days were defined with calculating around 80 % of the days between the lowest 10 % and highest 10 % daily delivery volume frequency days inside each hospital category (Table 1). Days with zero deliveries per day were not included in the calculations. Quiet days in the hospital category are represented when over resourcing of staffing may occur and busy days in the hospital category are represented when under resourcing of staffing may occur. Hospital level optimal days are represented when there are optimal staffing resources within the delivery unit.

For this study, three adverse maternal outcomes were chosen based on their prevalence in delivery units. Data availability and the results of previous literature also supported this decision. Including blood transfusions as a defined outcome was chosen as a surrogate for postpartum haemorrhage (PPH). PPH is one of the most severe maternal outcomes and is also listed as a considerable cause of maternal death during labour [14]. Definition of blood transfusions in this study includes interventions of red blood cell transfusion, thrombocytes and fresh frozen plasma during delivery or postpartum in a delivery unit. Maternal outcome is defined as MRP which also included diagnoses of a retained placenta. MRP is likely to be related to PPH and if it occurs, may need additional emergency obstetric care [15]. Maternal adverse outcome is defined as OASIS including information of third- and fourth- degree perineal tears. OASIS as widely used as a perinatal health indicator and is also associated with reflections of maternal morbidity [16, 17].

Bivariable analyses and multivariable regression analyses were utilized to analyse the association between the varying daily delivery volume (quiet days, optimal days, busy days) and adverse maternal outcomes (blood transfusion, MRP, OASIS). Prevalence, crude odds ratio (cOR) and adjusted OR (aOR) with 99 % confidence intervals (CIs) were defined. Multivariable regression analyses were chosen as an approach to take patient mix differences caused by patient flow into account. Statistical analyses were conducted within each hospital category (C1-C5) and the deliveries in each hospital categories served their own controls speculating the patient mix to be roughly similar despite of daily variation in delivery volume. Defined quiet days and busy days were compared to hospital category level optimal days and by that optimal days were set as a reference group in each hospital category. Due to multiple comparisons, associations were considered statistically significant if *p* -value was < 0.01. All analyses were conducted with SPSS statistical software (version 25).

To analyse the association between varying daily delivery volume and blood transfusions, all delivery modes were included in the analysis (vaginal delivery, vacuum assisted delivery, Caesarean delivery). Covariates were chosen and categorised as maternal age, parity, birth weight and delivery mode. To analyse associations between daily delivery volume and adverse maternal outcomes defined as MRP and OASIS, only vaginal deliveries were included in the analysis. Covariates for MRP were defined as categorised maternal age, parity and birth weight. Covariates for OASIS were defined as categorised maternal age, parity, birth weight, vacuum assisted delivery. Covariates were defined based on earlier literature and categorised by delivery mode (vaginal delivery, vacuum assisted delivery, caesarean section), maternal age (< 25, 25 to 34, ≥ 35 years), parity (nulliparous, multiparous), and birth weight (< 3000 g, 3000–3999 g, ≥ 4000 g).

## Results

Maternal and foetal characteristics are reported by varying daily delivery volume (quiet, optimal, busy days) in each hospital category in Table 2.

**Table 2:** Maternal characteristics during quiet, optimal and busy days by hospital categorisation

Hospital category	Maternal characteristics	Categorisation	Quiet day (n, %)	Optimal day (n, %)	Busy day (n, %)
C1	Delivery mode	Vaginal delivery	5509 (76.4)	34,254 (77.8)	3298 (78.9)**
		Vacuum assisted delivery	524 (7.3)	3317 (7.5)	294 (7.0)
		Caesarean section	1179 (16.3)	6485 (14.7)	588 (14.1)
	Parity	Nulliparous	2788 (38.7)	16,339 (37.1)	1513 (36.2)
	Age	< 25	1438 (19.9)	9225 (20.9)	886 (21.2)
		25–34	4465 (61.9)	27,039 (61.4)	2522 (60.3)
		≥ 35	1309 (18.2)	7792 (17.7)	772 (18.5)
	Birth weight	< 3000	909 (12.6)	5329 (12.1)	475 (11.4)
		3000–3999	5078 (70.4)	31,126 (70.7	2952 (70.6)
		≥ 4000	1224 (17.0)	7586 (17.2)	753 (18.0)
C2	Delivery mode	Vaginal delivery	10,875 (76.6)	104,208 (76.2)	10,990 (75.0)
		Vacuum assisted delivery	1237 (8.7)	11,984 (8.8)	1197 (8.2)***
		Caesarean section	2088 (14.7)	20,518 (15.0)	2475 (16.9)
	Parity	Nulliparous	5705 (40.2)	54,747 (40.0)	5781 (39.4)
	Age	< 25	2732 (19.2)	26,565 (19.4)	2815 (19.2)
		25–34	8938 (62.9)	86,463 (63.2)	9259 (63.1)
		≥ 35	2530 (17.8)	23,683 (17.3)	2588 (17.7)
	Birth weight	< 3000	1936 (13.6)	17,695 (12.9)	1913 (13.1)
		3000–3999	9854 (69.4)	95,316 (69.7)	10,120 (69.0)
		≥ 4000	2408 (17.0)	23,678 (17.3)	2625 (17.9)
C3	Delivery mode	Vaginal delivery	4022 (75.8)	31,734 (74.3)	4777 (72.7)***
		Vacuum assisted delivery	433 (8.2)	3743 (8.8)	556 (8.5)
		Caesarean section	848 (16.0)	7221 (16.9)	1240 (18.9)
	Parity	Nulliparous	2044 (38.5)	16,485 (38.6)	2583 (39.3)
	Age	< 25	1092 (20.6)	8310 (19.5)	1248 (19.0)
		25–34	3316 (62.5)	26,870 (62.9)	4184 (63.7)
		≥ 35	895 (16.9)	7518 (17.6)	1141 (17.4)
	Birth weight	< 3000	585 (11.0)	5110 (12.0)	863 (13.1)***
		3000–3999	3636 (68.6)	29,468 (69.1)	4569 (69.6)
		≥ 4000	1082 (20.4)	8096 (19.0)	1135 (17.3)
C4	Delivery mode	Vaginal delivery	7777 (80.2)	67,102 (76.3)	7992 (77.1)***
		Vacuum assisted delivery	796 (8.2)	8827 (10.0)	1155 (11.1)
		Caesarean section	1130 (11.6)	12,000 (13.6)	1215 (11.7)
	Parity	Nulliparous	4103 (42.2)	41,445 (47.0)	5115 (49.3)***
	Age	< 25	1182 (12.1)	11,331 (12.9)	1227 (11.8)***
		25–34	6428 (66.1)	57,858 (65.6)	6668 (64.3)
		≥ 35	2121 (21.8)	18,967 (21.5)	2472 (23.8)
	Birth weight	< 3000	1071 (11.0)	10,793 (12.3)	1401 (13.5)***
		3000–3999	6924 (71.4)	62,362 (70.9)	7371 (71.2)
		≥ 4000	1706 (17.6)	14,744 (16.8)	1587 (15.3)
C5	Delivery mode	Vaginal delivery	18,270 (74.2)	126,639 (74.0)	15,788 (73.1)***
		Vacuum assisted delivery	1865 (7.6)	14,560 (8.5)	1871 (8.7)
		Caesarean section	4478 (18.2)	29,889 (17.5)	3952 (18.3)
	Parity	Nulliparous	10,328 (42.0)	72,826 (42.6)	9620 (44.5)***
	Age	< 25	4530 (18.4)	27,935 (16.3)	3138 (14.5)***
		25–34	15,168 (61.6)	108,552 (63.4)	13,879 (64.1)
		≥ 35	4915 (20.0)	34,661 (20.3)	4620 (21.4)
	Birth weight	< 3000	4017 (16.3)	26,115 (15.3)	3334 (15.4)***
		3000–3999	16,694 (67.8)	115,890 (67.7)	14,708 (68.1)
		≥ 4000	3895 (15.8)	29,051 (17.0)	3565 (16.5)

*** *p* < 0.001, ** *p* < 0.01

C1 = < 1000 deliveries annually, C2 = 1000–1999 deliveries annually, C3 = 2000–2999 deliveries annually, C4 = ≥ 3000 deliveries annually, C5 = University hospitals

Busy days were associated with the increased need for blood transfusion in hospital category C2 and C5, where blood transfusion was needed 28 % (99 % CI 8–52 %) and 25 % (99 % CI 11 %-40 %) more often, respectively, compared to the need for blood transfusion during optimal days. In hospital category C3, the need for blood transfusion was 83 % (99 % CI 65–92 %) less during busy days compared to the need for blood transfusion during optimal days. Quiet days were associated with a decreased need for blood transfusion in hospital category C5, where blood transfusion was performed 22 % (99 % CI 10–31 %) less compared to optimal days. Varying daily delivery volume times were not associated with MRP in any hospital category. The association between varying delivery volume time periods and OASIS was noticed only in hospital category C5, where the occurrence of OASIS was 22 % (99 % CI 3–28 %) less during the quiet days compared to optimal days (Table 3).

**Table 3:** Prevalence, odds ratio (OR), adjusted OR (aOR), 99 % confidence intervals (CIs) and maternal outcomes during varying delivery volume time periods (quiet, optimal, busy) in each hospital category

Hospital category	Population	Maternal outcome	Quiet day	Optimal day	Busy day
C1	All deliveries (*n* = 55 448, 100.0 %)	Blood transfusion (n, %)	80 (1.1)	439 (1.0)	47 (1.0)
		OR (99 % CI)	1.11 (0.81–1.53)	Ref.	1.13 (0.76–1.68)
		aOR (99 % CI)	1.11 (0.80–1.51)	Ref.	1.13 (0.76–1.69)
	Vaginal Deliveries (*n* = 47 196, 85.1 %)	Manual removal of placenta (n, %)	134 (2.2)	822 (2.2)	71 (2.0)
		OR (99 % CI)	1.02 (0.78–1.30)	Ref.	0.90 (0.65–1.24)
		aOR (99 % CI)	0.98 (0.78–1.27)	Ref.	0.90 (0.65–1.25)
		OASIS (n, %)	57 (0.9)	287 (0.8)	28 (0.8)
		OR (99 % CI)	1.24 (0.85–1.80)	Ref.	1.02 (0.61–1.70)
		aOR (99 % CI)	1.24 (0.85–1.80)	Ref.	1.04 (0.62–1.74)
C2	All deliveries (*n* = 165 573, 100.0 %)	Blood transfusion (n, %)	174 (1.2)	1906 (1.4)	263 (1.8)***
		OR (99 % CI)	0.88 (0.72–1.08)	Ref.	1.29 (1.09–1.53)***
		aOR (99 % CI)	0.87 (0.71–1.07)	Ref.	1.28 (1.08–1.52)***
	Vaginal deliveries (*n* = 140 491, 84.9 %)	Manual removal of placenta (n, %)	215 (1.8)	2026 (1.7)	199 (1.6)
		OR (99 % CI)	1.02 (0.86–1.23)	Ref.	0.94 (0.77–1.13)
		aOR (99 % CI)	1.01 (0.84–1.22)	Ref.	0.94 (0.77–1.14)
		OASIS (n, %)	88 (0.7)	1011 (0.9)	93 (0.8)
		OR (99 % CI)	0.83 (0.63–1.11)	Ref.	0.88 (0.66–1.16)
		aOR (99 % CI)	0.84 (0.63–1.12)	Ref.	0.89 (0.67–1.18)
C3	All deliveries (*n* = 54 574, 100.0 %)	Blood transfusion (n, %)	78 (1.5)	497 (1.2)	13 (0.2)***
		OR (99 % CI)	1.27 (0.92–1.74)	Ref.	0.17 (0.08–0.35)***
		aOR (99 % CI)	1.28 (0.92–1.73)	Ref.	0.17 (0.08–0.35)***
	Vaginal deliveries (*n* = 45 265, 82.9 %)	Manual removal of placenta (n, %)	67 (1.5)	626 (1.8)	77 (1.4)
		OR (99 % CI)	0.85 (0.61–1.19)	Ref.	0.82 (0.60–1.12)
		aOR (99 % CI)	0.86 (0.62–1.21)	Ref.	0.80 (0.58–1.09)
		OASIS (n, %)	34 (0.8)	237 (0.7)	41 (0.8)
		OR (99 % CI)	1.14 (0.71–1.84)	Ref.	1.15 (0.74–1.78)
		aOR (99 % CI)	1.15 (0.72–1.86)	Ref.	1.17 (0.75–1.82)
C4	All deliveries (*n* = 108 254, 100.0 %)	Blood transfusion (n, %)	403 (4.1)	3353 (3.8)	374 (3.6)
		OR (99 % CI)	1.09 (0.95–1.25)	Ref.	0.95 (0.82–1.09)
		aOR (99 % CI)	1.14 (0.99–1.31)	Ref.	0.93 (0.80–1.07)
	Vaginal deliveries (*n* = 93 649, 86.5 %)	Manual removal of placenta (n, %)	177 (2.1)	1611 (2.1)	185 (2.0)
		OR (99 % CI)	0.97 (0.80–1.20)	Ref.	0.95 (0.78–1.17)
		aOR (99 % CI)	1.00 (0.81–1.23)	Ref.	0.92 (0.75–1.12)
		OASIS (n, %)	161 (1.9)	1291 (1.7)	165 (1.8)
		OR (99 % CI)	1.10 (0.89–1.38)	Ref.	1.06 (0.86–1.32)
		aOR (99 % CI)	1.20 (0.96–1.50)	Ref.	1.04 (0.84–1.29)
C5	All deliveries (*n* = 217 398, 100.0 %)	Blood transfusion (n, %)	420 (1.7)***	3710 (2.2)	597 (2.8)***
		OR (99 % CI)	0.78 (0.69–0.90)***	Ref.	1.28 (1.14–1.44)***
		aOR (99 % CI)	0.78 (0.69–0.90)***	Ref.	1.25 (1.11–1.40)***
	Vaginal deliveries (*n* = 178 993, 82.3 %)	Manual removal of placenta (n, %)	272 (1.4)	1921 (1.4)	263 (1.4)
		OR (99 % CI)	0.99 (0.84–1.17)	Ref.	1.10 (0.92–1.30)
		aOR (99 % CI)	1.01 (0.85–1.15)	Ref.	1.06 (0.90–1.26)
		OASIS (n, %)	154 (0.8)***	1475 (1.0)	209 (1.2)
		OR (99 % CI)	0.73 (0.59–0.91)***	Ref.	1.14 (0.94–1.37)
		aOR (99 % CI)	0.78 (0.62–0.97)**	Ref.	1.10 (0.91–1.33)

*** *p* < 0.001

** *p* < 0.01

Maternal outcomes adjusted (aOR) with:

1) Blood transfusion and maternal age (categorical), parity (nulliparous or multiparous), birth weight (categorical), delivery mode (categorical)

2) MRP and maternal age (categorical), parity (nulliparous or multiparous), birth weight (categorical)

3) OASIS and maternal age (categorical), parity (nulliparous or multiparous), birth weight (categorical), vacuum extraction assisted delivery (vacuum extraction yes/no)

## Discussion

The busy day effect has been studied on selected adverse maternal outcomes in different sized hospital categories. The busy day effect made statistical differences between the need for blood transfusions and the occurrence of OASIS rates but not in the performance of MRP. The overall need for blood transfusion among all deliveries ranged from 1 to 4 %, being lowest at small hospitals (C1) and highest at large non-university hospitals (C4). Interestingly, university hospitals were in between these two categories. Busy days appeared to increase the need for blood transfusion in C2 and university hospitals by 28 % and 25 %, respectively, whereas 22 % less blood transfusions were needed at university hospitals during quiet days. Unexpectedly, in hospital category C3, 83 % less blood transfusions were needed during busy days compared to optimal days. With respect to OASIS rates, the only significant change was related to daily delivery volume variation that was noticed at university hospitals where the OASIS rate declined by 22 % during quiet days.

The virtually unchanging rate of MRP independent of unit size or workload was not only an expected finding but also a result that helps to validate the findings of the study. Accordingly, the increasing need of blood transfusion during busy days was also expected, but the fact that it occurred in small non-university (C2) and university hospitals only, was interesting. It can be speculated that not all diagnostic or preventive measures [18] against haemorrhage were performed or were not adequately performed during the busiest times, resulting in the need of blood transfusion. Unexpectedly, in middle-size hospitals (C3), significantly fewer transfusions were needed when the workload was high.

Statistically it can be assumed that up to 40 cases requiring blood transfusions are missing from that group, and even though the data and the analysis has been verified, this appears to be the case. It is unlikely that cases with significant PPH with the need of blood transfusion would go unnoticed or would not be registered since in every other hospital category independently of the workload, the numbers seem to be consistent. Therefore, it is speculated that hospitals particularly in this size category (C3), when heavily loaded, tend to transfer patients to university hospitals when the risk of PPH is recognized. There is no evidence of this in the MBR since it lacks such referral data, however, it is known that C3 hospitals have only one obstetrician on call at hospital over nights and weekends. Consequently, their surgical capacity and blood bank availability appear to be the limiting factor under busy days, whereas in large non-university and university hospitals with adequate staffing and multiple surgical teams, resourcing is not as critical as in smaller units. Furthermore, in category C3 and smaller hospitals, interventional radiology is not available outside regular working hours, if at all.

Quiet days appeared to be beneficial in decreasing the OASIS rate and need for blood transfusion at university hospitals but not in other hospital categories. This result can be associated with midwifery- led work and hospital-based nursing culture in teaching hospitals where the chances to consult more experienced health care professionals may be easier when the overall workload is low.

Strength of this study is based on the data collection from MBR, that has previously demonstrated to be reliable and informative for this kind of population-based register evaluation [19, 20]. The large data sample made it possible to define different varying daily delivery volume time periods (quiet, optimal, busy days) and hospital categorisation. The large data set also enabled more fulsome statistical analysis and the use of case-mix evaluation improved the reliability.

Weaknesses of this study include the definition of varying delivery volume time periods as quiet, optimal and busy days, in different sized hospital categories. Calculations were based on estimates and were not exact or the same in all different hospital categories, due to the varying number of deliveries per day. Calculations were performed on delivery frequency per day which simplifies natural delivery processes. Deliveries can take place 24/7 and birth date links the case only to the end of the process. Another weakness is due to limited information on the hospital staffing levels and expertise in each hospital category and more detailed hospital level data.

## Conclusions

These results in this kind of study setting are for our best knowledge novel. The findings of this study do not identify specific patterns to busy days in respect to adverse maternal outcomes that are defined as manual removal of placenta or OASIS, but both quiet and busy days seem to be associated with increased or decreased use of blood transfusion in different sized delivery units. Quiet days are also associated with decreased number of OASIS. Otherwise varying delivery volume is a poor indicator of maternal adverse outcomes. Quiet days may be beneficial in university teaching hospitals where adequate time for consultation and for experienced help may play a role in the prevention of blood loss requiring blood transfusion and OASIS.

The study of the busy day effect is a new way to assess quality indicators as maternal outcome measures in the field of maternity care. Results of this study can add understanding of a delivery unit´s capacity to work under stress and to help resource units adequately. More research is still needed to confirm these findings and if repeated, the results suggest that quality should be routinely assessed separately for busy days to make sure that the unit is able to respond to high patient volumes and maintain expected levels of care.

## Data Availability

The datasets generated and/or analysed during the current study are not publicly available due to the nature of the register study and guidance of the Finnish national data protection legislation, but statistical tables are available from the corresponding author on reasonable request.

## Abbreviations

MBR: Medical Birth Register
MRP: Manual Removal of Placenta
OASIS: Obstetric Anal Sphincter Injuries
PPH: Post Partum Haemorrhage

## References

[1] Snowden JM, Backes Kozhimannil K, Muoto I, Caughey AB, McConnell KJ (2017). A “busy day” effect on perinatal complications of delivery on weekends: a retrospective cohort study. BMJ Qual Saf.

[2] Euro-Peristat Project. European Perinatal Health Report. Health and Care of Pregnant Women and Babies in Europe in 2010. https://www.europeristat.com/index.php/our-indicators/euro-peristat-perinatal-health-indicators-2010.html [Accessed 23 Apr 2020].

[3] Friedman AM, Ananth CV, Huang Y, D’Alton ME, Wright JD. Hospital delivery volume, severe obstetrical morbidity, and failure to rescue. Am J Obstet Gynecol. 2016;215(6):795. e1-795.e14 . 10.1016/j.ajog.2016.07.039 PMC5124527 27457112

[4] Bozzuto L, Passarella M, Lorch S, Srinivas S (2019). Effects of Delivery Volume and High-Risk Condition Volume on Maternal Morbidity Among High-Risk Obstetric Patients. Obstet Gynecol.

[5] Kyser KL, Lu X, Santillan DA, et al. The association between hospital obstetrical volume and maternal postpartum complications. Am J Obstet Gynecol. 2012;207(1):42. e1-42.e17. 10.1016/j.ajog.2012.05.010 PMC4362705 22727347

[6] Aubrey-Bassler FK, Cullen RM, Simms A (2019). Population-based cohort study of hospital delivery volume, geographic accessibility, and obstetric outcomes. Int J Gynaecol Obstet.

[7] Snowden JM, Cheng YW, Emeis CL, Caughey AB. The impact of hospital obstetric volume on maternal outcomes in term, non-low-birthweight pregnancies. Am J Obstet Gynecol. 2015;212(3):380. e1-380.e11. 10.1016/j.ajog.2014.09.026 PMC4346499 25263732

[8] Karalis E, Tapper AM, Gissler M, Ulander VM (2018). The impact of increased number of low-risk deliveries on maternal and neonatal outcomes: A retrospective cohort study in Finland in 2011–2015. Eur J Obstet Gynecol Reprod Biol.

[9] Clapp MA, James KE, Kaimal AJ. The effect of hospital acuity on severe maternal morbidity in high-risk patients. Am J Obstet Gynecol. 2018;219(1):111. e1-111.e7. 10.1016/j.ajog.2018.04.015 29673571

[10] de Graaf JP, Ravelli AC, Visser GH (2010). Increased adverse perinatal outcome of hospital delivery at night. BJOG.

[11] Lyndon A, Lee HC, Gay C, Gilbert WM, Gould JB, Lee KA. Effect of time of birth on maternal morbidity during childbirth hospitalization in California. Am J Obstet Gynecol. 2015;213(5):705. e1-705.e11. 10.1016/j.ajog.2015.07.018 PMC4631702 26196454

[12] Bailit JL, Grobman WA, Rice MM, et al. Risk-adjusted models for adverse obstetric outcomes and variation in risk-adjusted outcomes across hospitals. Am J Obstet Gynecol. 2013;209(5):446. e1-446.e30. 10.1016/j.ajog.2013.07.019 PMC4030746 23891630

[13] International statistical classification of (2010). diseases and related health problems, tenth revision.

[14] Troop P, Goldacre M, Mason A, Cleary R, editors. Health Outcome Indicators: Normal Pregnancy and Childbirth. Report of a working group to the Department of Health. Oxford: National Centre for Health Outcomes Development; 1999. http://nchod.uhce.ox.ac.uk/pregnancy.pdf. [Accessed 25 May 2020].

[15] OECD (2013). Health at a Glance 2013: OECD Indicators.

[16] Sibanda T, Fox R, Draycott TJ, Mahmood T, Richmond D, Simms RA (2013). Intrapartum care quality indicators: a systematic approach for achieving consensus. Eur J Obstet Gynecol Reprod Biol.

[17] Lazzaretto E, Nespoli A, Fumagalli S, Colciago E, Perego S, Locatelli A (2018). Intrapartum care quality indicators: a literature review. Minerva Ginecol.

[18] Merriam AA, Wright JD, Siddiq Z (2018). Risk for postpartum hemorrhage, transfusion, and hemorrhage-related morbidity at low, moderate, and high volume hospitals. J Matern Fetal Neonatal Med.

[19] Gissler M, Shelley J. 2002. Quality of data on subsequent events in a routine Medical Birth Register. Med Inform 2002;27(1):33–38. 10.1080/14639230110119234 12509121

[20] Gissler M, Teperi J, Hemminki E, Meriläinen J (1995). Data quality after restructuring a national medical registry. Scand J Public Health.

